# Spontaneous Mutation in the Movement Protein of Citrus Leprosis Virus C2, in a Heterologous Virus Infection Context, Increases Cell-to-Cell Transport and Generates Fitness Advantage

**DOI:** 10.3390/v13122498

**Published:** 2021-12-13

**Authors:** Mikhail Oliveira Leastro, David Villar-Álvarez, Juliana Freitas-Astúa, Elliot Watanabe Kitajima, Vicente Pallás, Jesús Ángel Sánchez-Navarro

**Affiliations:** 1 Unidade Laboratorial de Referência em Biologia Molecular Aplicada, Instituto Biológico, São Paulo 04014-900, Brazil; juliana.astua@embrapa.br; 2 Instituto de Biología Molecular y Celular de Plantas, Universidad Politécnica de Valencia-Consejo Superior de Investigaciones Científicas (CSIC), 46022 Valencia, Spain; davilal@posgrado.upv.es (D.V.-Á.); vpallas@ibmcp.upv.es (V.P.); 3 Embrapa Mandioca e Fruticultura, Cruz das Almas 70770-901, Brazil; 4 Departamento de Fitopatologia e Nematologia, Escola Superior de Agricultura Luiz de Queiroz, Universidade de São Paulo, Piracicaba 13418-900, Brazil; ewkitaji@usp.br

**Keywords:** viral fitness, cilevirus, alfalfa mosaic virus system, movement protein, amino acid mutation

## Abstract

Previous results using a movement defective alfalfa mosaic virus (AMV) vector revealed that citrus leprosis virus C (CiLV-C) movement protein (MP) generates a more efficient local movement, but not more systemic transport, than citrus leprosis virus C2 (CiLV-C2) MP, MPs belonging to two important viruses for the citrus industry. Here, competition experiment assays in transgenic tobacco plants (P12) between transcripts of AMV constructs expressing the cilevirus MPs, followed by several biological passages, showed the prevalence of the AMV construct carrying the CiLV-C2 MP. The analysis of AMV RNA 3 progeny recovered from P12 plant at the second viral passage revealed the presence of a mix of progeny encompassing the CiLV-C2 MP wild type (MP^WT^) and two variants carrying serines instead phenylalanines at positions 72 (MP^S72F^) or 259 (MP^S259F^), respectively. We evaluated the effects of each modified residue in virus replication, and cell-to-cell and long-distance movements. Results indicated that phenylalanine at position 259 favors viral cell-to-cell transport with an improvement in viral fitness, but has no effect on viral replication, whereas mutation at position 72 (MP^S72F^) has a penalty in the viral fitness. Our findings indicate that the prevalence of a viral population may be correlated with its greater efficiency in cell-to-cell and systemic movements.

## 1. Introduction

In the course of evolution, plant viruses have developed a special class of proteins specialized in viral transport, called movement proteins (MPs) [1]. These MPs act in sync with host factors generating different pathways for virus transport addressed to overcome the plasmodesma (PD) barrier, which corresponds to specialized channels in the plant cell wall implicated in regulating the passage of small molecules between the cytoplasm of neighboring cells [2,3]. The MPs show the capacity to transport viral ribonucleoprotein complexes (RNPs) or viral particles from the site of virus replication and assembly into the cell periphery and through the PD, thus enabling viral cell-to-cell and long-distance transport [4,5,6,7]. Viral MPs have been organized into different groups based on sequence homology and predicted secondary structures [4,6]. The 30K MP superfamily includes MPs from both RNA and DNA viruses belonging to more than 20 genera, which show different movement strategies [5,8]. Members of this family have few conserved motifs in their amino acid sequence; the main one consists of a core central structure with seven predicted ß-strands flanked by α-helices on each end [6,8]. Furthermore, the 30K superfamily encompasses both tubule-forming and non-tubule-forming proteins [2,6,7,8,9,10,11]. Remarkably, it has been demonstrated that the MPs of tubule-forming viruses are capable of transporting viral ribonucleoprotein complexes in the alfalfa mosaic virus (AMV) model system [12,13].

The citrus leprosis complex represents the major viral disease affecting citrus orchards in the American continent. Citrus leprosis-associated viruses, transmitted by mite vectors of the genus *Brevipalpus,* exhibit significant differences in their genomic organization and the cellular infection process. Citrus leprosis virus C (CiLV-C) and citrus leprosis virus C2 (CiLV-C2) belong to the genus *Cilevirus*, family *Kitaviridae,* have a positive single-stranded RNA and replicate in the cell cytoplasm [14]. Although CiLV-C is considered the most devastating virus infecting citrus orchards in American countries [15], this situation is different in Colombia, where CiLV-C2 is prevalent in citrus orchards [14,16,17,18]. As reviewed by Leastro et al. [19], CiLV-C2 RNA 1 and RNA 2 present a nucleotide identity of 58 and 50%, respectively, compared to the corresponding CiLV-C RNA sequence, and their MPs present 51% of amino acid sequence identity [16].

Mutations in viral MPs can not only affect functional aspects implicated in virus transport but also can play important roles in symptom development, host susceptibility, virulence, and pathogenicity [20,21,22,23,24,25,26,27,28]. Interestingly, single amino acid mutations have been observed to affect either cell-to-cell or systemic movement [22,25,26,29,30].

Cilevirus MPs belong to the 30K superfamily [31,32,33] and their role in the viral movement has recently been described [34]. In a previous study about cileviruses MP functionality using an AMV chimeric construct expressing the CiLV-C and CiLV-C2 MPs, it was observed that CiLV-C MP generated a more efficient local movement than the CiLV-C2 MP, whereas an inverse correlation was observed for systemic spread [34].

In the present study, using the cilevirus MPs as a model for virus fitness study, we advance our knowledge about plant virus evolution and adaptation. We demonstrated that the AMV construct carrying the CiLV-C2 MP accumulated a higher amount of AMV RNA than construct carrying CiLV-C MP, indicating that the better efficiency in local movement was not associated with greater viral accumulation. Viral fitness analysis demonstrated that the viral AMV population harboring CiLV-C2 MP prevails over the population expressing the CiLV-C MP in transgenic *Nicotiana tabacum* expressing the AMV P1 and P2 replicase subunits (P12 plants) [35]. Remarkably, P12 plants infected with the AMV derivative carrying the CiLV-C2 MP revealed the presence of a mix of progeny encompassing the CiLV-C2 MP wild type (MP^WT^) and two MP mutants showing the exchange of serines by phenylalanines at positions 72 or 259 (MP^S72F^ and MP^S259F^). We evaluated the effects of each modified residue in virus replication, and cell-to-cell and long-distance movements on AMV infection context. Results revealed that a single mutation corresponding to the presence of a phenylalanine at position 259 generates more efficient cell-to-cell transport than MP^S72F^ and MP^WT^ constructs, but it had no effect on vRNA (viral RNA) accumulation and viral systemic movement, even though in the natural infection context the cileviruses are not able to reach their hosts systemically [14]. On the viral fitness analyses, our findings suggest that the prevalence of a viral population may be correlated with its greater efficiency in movement.

## 2. Materials and Methods

### 2.1. DNA Manipulation

For the cell-to-cell movement assay, a modified infectious AMV cDNA 3 clone (pGFP/A255/CP) [36], which expresses the green fluorescent protein (GFP), was used to exchange the 255 amino acids (aa) of the AMV MP gene with the corresponding MP of CiLV-C (strain CRD), wild type (WT) CiLV-C2 (strain L147V1) and the mutated versions of CiLV-C2 MP where a serine residue was replaced by phenylalanine at position 72 (MP^S72F^) or 259 (MP^S259F^). The genes were amplified from total RNA extracted from infected citrus leaves and fruits or transgenic *N. tabacum* P12 plants that constitutively express the AMV polymerase proteins P1 and P2 [35] with RiboZol reagent (VWR Life Science, Canadian), following the manufacturer’s instructions. The P12 plants permit to work only with the AMV RNA 3 or its derivatives, simplifying the analysis. The correspondent viral genes were generated from the One-Step RT-PCR system (Thermo Fisher Scientific, MA, USA), following the manufacturer’s instructions, with specific primers containing the restriction sites *Nco*I and *Nhe*I compatible with the AMV system. The resultant heterologous proteins were fused with the C-terminal 44 amino acids (A44) of the AMV MP, which are required for a compatible interaction with the cognate AMV capsid protein (CP) [37].

For viral fitness and viral movement systemic analyses, the MP genes aforementioned and their mutated versions, CiLV-C2 MP (MP^S72F^ and MP^S259F^), were introduced in a chimeric infectious AMV cDNA 3 clone (pAL3NcoP3), lacking the GFP gene, since the RNA 3 derivatives carrying the GFP reporter gene do not support systemic movement in P12 plants [36].

### 2.2. Inoculation of P12 Plants and Protoplasts for Analysis of Cell-to-Cell Movement, Systemic Spread, or RNA Accumulation

For the synthesis of the RNA transcripts, all AMV 3 cDNA constructs were amplified first using specific primer pairs targeting the T7 promoter and the AMV 3′ end. The generated amplicons were used directly as templates for in vitro transcription with T7 RNA polymerase (Takara Bio Inc, Shiga, Japan). At least three (unless specifically noted otherwise) transgenic *N. tabacum* P12 plants were grown and inoculated with RNA transcripts, as previously described [38]. The foci images in P12 plants were taken with the aid of a stereomicroscope (MZZ16F Leica, Wetzlar, Germany) with the area of infection foci measured at two- and three-days post-inoculation (dpi), using ImageJ version 2.0cr software. The graphs represent the average of the area in mm^2^ of 40 independent infection foci from each construct. Each construct was inoculated on two plants with two (for systemic analysis) or three (for cell-to-cell analysis) leaves per plant, and 15 µL (~3 µg) of the transcription mixture was used as inoculum per leaf.

For analysis of AMV RNA accumulation, protoplasts were extracted from P12 leaves and 2.5 × 10^5^ protoplasts were inoculated by the polyethylene glycol method [39] with the AMV transcript mixture carrying the CiLV-C MP, CiLV-C2 MP^WT^ and the mutated versions. Protoplast preparation was performed in duplicate for each sample.

### 2.3. Northern Blot and Tissue Print Assays

Total RNA was extracted from P12 protoplasts at 16 h post-inoculation using RiboZol reagent (VWR Life Science, Edmonton, AB, Canada). The RNAs were electrophoresed through a formaldehyde-denatured gel and transferred to positively charged nylon membranes (Roche, Mannheim, Germany). RNAs were fixed to the membranes with a UV cross-linker (700 × 100 µJ/cm^2^). Hybridization and detection were conducted as previously described [40] using a dig-riboprobe (Roche, Mannheim, Germany) complementary to the 3′ UTRs of the AMV RNA 3.

Tissue-printing analyses were performed by transversal sections of the corresponding petiole from inoculated (I) and upper (U) P12 leaves at 14 days post-inoculation (dpi), as indicated previously [41]. All results shown from tissue-printing are representatives of three independent assays. The hybridization and detection were conducted as mentioned above.

### 2.4. Statistical and In Silico Analysis

The Student’s *t*-test and one-way ANOVA were performed to determine the significant differences between MPs experiments analyzing cell-to-cell efficiency and AMV RNA accumulation. Significance values with *p* ≤ 0.05 or no significant difference were displayed in the graphs. The Northern blot graph presented represents the total accumulation of the RNAs corresponding to the average of two Northern blot analyses from two independent experiments, which error bars indicate the standard deviation [42]. The bands were quantified using ImageJ version 2.0cr software with the ISAC plugin.

Alignment of the MPs was performed using the PROMALS3D program [43], which annotates secondary structural features using the PSIPRED algorithm through consensus predictions [44]. The GenBank accession numbers of the cilevirus MPs used for this analysis are CiLV-C strain CRD (YP_654542.1), CiLV-C strain SJP (AKJ79137.1), CiLV-C2 strain L147V1 (AGE82893.1). The MP of tentative cilevirus passion fruit green spot virus (PfGSV) strain Snp1 (MK804172.1) was also used in this analysis.

### 2.5. Viral Fitness Assay

For the competition assays, we first balanced the concentration of AMV RNA 3 transcripts carrying the heterologous CiLV-C and CiLV-C2 MPs. The quantification was performed with agarose gel electrophoresis using an RNA ladder (RiboRuler High Range RNA Ladder, Thermo Scientific, Waltham, MA, USA) and several dilutions of the transcribed RNAs as Leastro et al. [45]. Next, two transgenic P12 leaves per plant and two plants for each pair combination were mechanically inoculated with RNA 3 transcripts harboring the respective heterologous MPs at the same concentration. The first transgenic tobacco plants were inoculated with a mix of transcripts containing the MPs, then three serial passages, each at 15 days post-infection (dpi), were performed using extracts of the systemic leaves as inocula. Independent experiments were repeated three times (six plants per serial passage for each pair combination inoculated, totaling 18 plants after the third passage). The prevalent isolate after the successive passages was determined by visualization of the correspondent MP DNA band in agarose gel electrophoresis through RT-PCR analysis using the SuperScript III One-Step RT-PCR System with Platinum *Taq* High Fidelity DNA Polymerase (Thermo Fischer, MA, USA), and using specific primers for the complete CiLV-C MP (fwd 5′-aaccatggctctttctaccaataacaattc-3′/rev 5′-aaaagctagcttattcgcttgtagaagttg-3′) and CiLV-C2 MP (fwd 5′-aaccatggtgtctgttggtgctgacg-3′/rev 5′-aaaagctagcctaagaaagcgttggtccatcg-3′). For competitive assay between CiLV-C2 MP^WT^ and MP^S72F^ or MP^S259F^, the prevalent isolate was determined by direct sequencing (Sanger sequencing method) of the RT-PCR amplicons using primers specific for regions in AMV RNA 3 flaking the MP gene. The direct sequencing was performed from two P12 plants for each combination pair of AMV mix transcripts carrying the CiLV-C2^WT^ and mutated versions until the third passage.

## 3. Results

### 3.1. Constructs Harboring CiLV-C2 MP Prevail over CiLV-C MP Constructs in Competition Experiments

**Figure 1:**
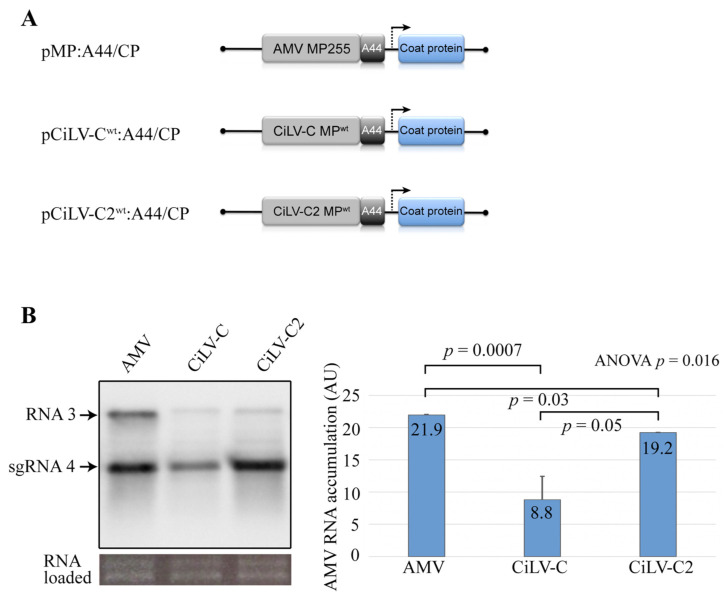
Northern blot analysis of the accumulation of alfalfa mosaic virus (AMV) RNA carrying citrus leprosis virus C (CiLV-C) and citrus leprosis virus C2 (CiLV-C2) movement proteins (MPs) in transgenic *Nicotiana tabacum* (P12) protoplasts. (**A**) The schematic representation shows the pAL3NcoP3 AMV RNA 3 construct [36] and its derivatives carrying the heterologous MPs fused to the C-terminal 44 residues of the AMV MP (A44), used in this assay. The open reading frames, represented by large boxes, correspond to the MP, and the coat protein (CP). The short box corresponds to the A44, meanwhile, arrow represents subgenomic promoter. (**B**) Northern blot showing accumulation of AMV RNAs in P12 protoplasts at 16 h post-inoculation, using a dig-riboprobe complementary to the 3′ UTR of the AMV RNA 3. Transcripts correspond to the AMV RNA 3 derivative carrying the CiLV-C MP, CiLV-C2 MP, and AMV MP (positive control) proteins. The localization of RNA 3 and subgenomic RNAs (sgRNA 4) are indicated. The graph represents the total accumulation of AMV RNA, carrying the constructs AMV MP, CiLV-C MP, and CiLV-C2 MP from the average of two independent experiments. The bands were quantified using the ImageJ version 2.0cr software with the ISAC plugin and error bars represent standard deviation. Statistical analysis was performed using Student’s *t*-test and one-way ANOVA. *p* values less than 0.05 (typically ≤ 0.05) are statistically significant. *p*-values were obtained from pairwise comparisons between treatments or among the three groups.

We previously observed that the expression of the CiLV-C and CiLV-C2 MPs using AMV RNA 3 chimeric constructs in transgenic *N. tabacum* P12 plants revealed that CiLV-C MP generated a more efficient local movement but not a more systemic transport than the CiLV-C2 MP [34] (Supplementary Figure S1). To better understand this phenomenon, we quantified the AMV RNA accumulation on P12 protoplasts at 16 h post-transfection. Northern blot analysis revealed that the AMV derivative (Figure 1A) carrying the CiLV-C2 MP accumulated (*p* values ≤ 0.05) a higher amount of AMV RNA than constructs carrying CiLV-C MP (Figure 1B). These results indicate that viral accumulation does not correlate with the efficiency of cell-to-cell movement.

**Figure 2:**
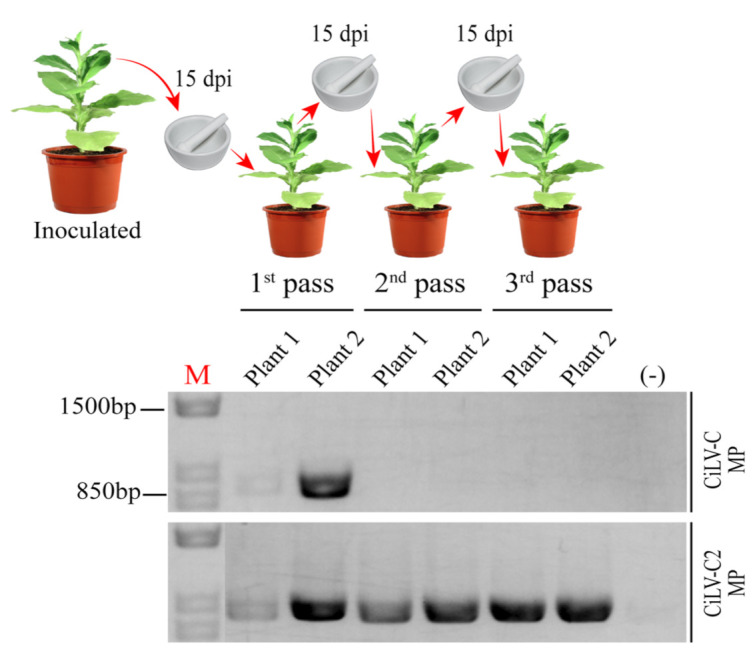
Competition analysis between alfalfa mosaic virus constructs expressing the MPs of CiLV-C and CiLV-C2 in transgenic *N. tabacum* plants. Co-inoculation of *N. tabacum* P12 plants with an infectious mixture containing equivalent amounts of AMV RNA 3 derivatives transcripts carrying the MP genes of the CiLV-C and CiLV-C2. These AMV RNA 3 derivative constructs are the same as shown in Figure 1A (pMP-CiLV-C^WT^:A44/CP and pMP-CiLV-C2^WT^:A44/CP). Schematic representation of three serial passages at 15 day intervals using extracts of the systemic leaves as inoculum is presented. The prevalent virus for each passage was determined by RT-PCR analysis using specific primers for each MP gene. The presence or absence of the correspondent MP DNA band in agarose gel electrophoresis is shown through RT-PCR analysis of two plants for each viral passage. The assay was repeated three times. (-) corresponds to a healthy plant. M = GeneRuler 1 kb DNA ladder marker. The marker band sizes of 1.5 kbp and 850 bp are indicated.

To gain additional insight about the movement performance of these proteins, we performed a competition assay between CiLV-C MP and CiLV-C2 MP AMV chimeric constructs. To do this, P12 plants were co-inoculated with an infection mixture containing equivalent amounts of the AMV RNA 3 transcript derivatives carrying the CiLV-C and CiLV-C2 MPs. Using extracts of the systemic leaves as inoculum, the prevalent AMV chimeric population present in the infected systemic tissue was determined by RT-PCR analysis using specific primers for CiLV-C and CiLV-C2 MPs during three serial passages at 15 day intervals. Viral inoculum for serial passages corresponded to a mixture of systemic leaves. In the first viral passage, we observed the presence of both cilevirus MP genes in systemic infected P12 leaves (Figure 2). However, from the second pass onwards only the presence of the CiLV-C2 MP gene (Figure 2) was identified, indicating the prevalence of the AMV population expressing the CiLV-C2 MP over the CiLV-C MP. These findings were consistent in all three independent experiments.

### 3.2. The New Variants of the CiLV-C2 MP Carrying Phenylalanines at 72 and 259 Positions Do Not Increase Significantly Viral Replication

To further characterize the viral progeny present in the P12 plants from the competition assay mentioned above, the MP genes were amplified, cloned into a modified pUC18 plasmid [36], and the nucleotide sequence of ten clones was determined.

**Figure 3:**
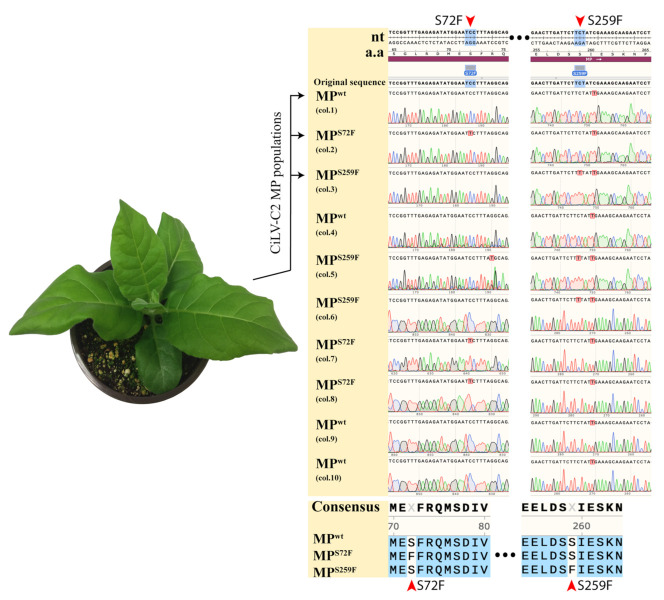
CiLV-C2 MP variants recovered from P12 plant in viral fitness study. P12 plant infected with the AMV population expressing the CiLV-C2 MP is visualized at the left of the figure. P12 images are representative of 18 infected plants. At the right of the figure is presented chromatograms from ten clones recovered from the P12 plant at the second viral passage. The exchange of serines for phenylalanines at positions 72 (MP^S72F^) and 259 (MP^S259F^) is highlighted by red arrows. Below the chromatograms are displayed amino acids consensus sequences of MPs revealing mutations 72 (MP^S72F^) and 259 (MP^S259F^) highlighted by red arrows. Chromatograms and sequence alignment were performed using the software SnapGene v. 5.0.8.

Complete sequences of CiLV-C2 MP from P12 plants at the second viral passage showed the presence of the wild type gene (MP^WT^) in 40% of clones (four out of ten colonies), but also nucleotide alteration leading to changes in deduced amino acid residues, resulting in the exchange of serine for phenylalanine either at position 72 (MP^S72F^) (30% of clones, three out of ten) or position 259 (MP^S259F^) (30% of clones, three out of ten), indicating the presence of a mix infection with at least three different MP gene versions (Figure 3). These mutations were identified in one line of the P12 plants at the second viral passage.

**Figure 4:**
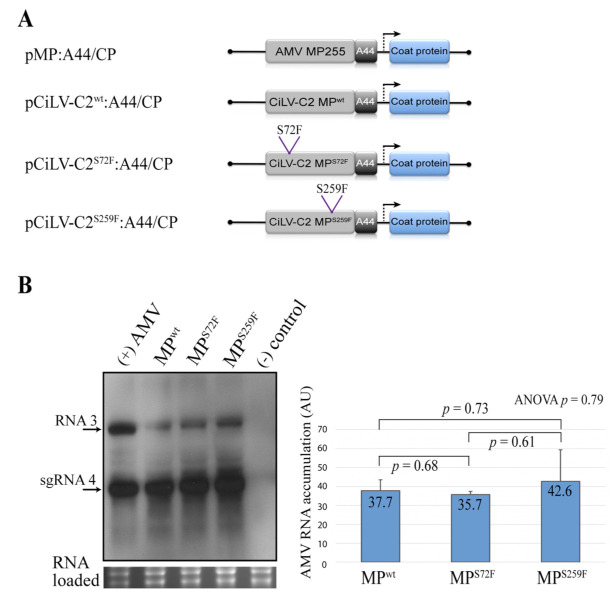
Northern blot analysis of the accumulation of AMV RNA in P12 protoplasts. (**A**) The schematic representation shows the pAL3NcoP3 AMV RNA 3 construct [36], lacking the GFP gene and its derivatives carrying the heterologous CiLV-C2 MPs wild type (WT) and mutated versions (MP^S72F^ and MP^S259F^) fused to the C-terminal 44 residues of the AMV MP (A44), used in this assay. (**B**) Northern blot showing accumulation of AMV RNAs in P12 protoplasts at 16 h post-inoculation, using a dig-riboprobe complementary to the 3′ UTR of the AMV RNA 3. Transcripts correspond to the AMV RNA 3 derivative carrying the CiLV-C2 MP^WT^, mutated (MP^S72F^ and MP^S259F^) and AMV MP (positive control) proteins. Negative control (-) corresponds to the non-infected P12 protoplasts. The localization of RNA 3 and subgenomic RNAs (sgRNA 4) are indicated. The graph represents the total accumulation of AMV RNA, carrying the constructs CiLV-C2 MP^WT^, MP^S72F^, and MP^S259F^ from the average of two independent experiments. The bands were quantified using the ImageJ v. 2.0cr software with the ISAC plugin and error bars represent standard deviation. Statistical analyses were performed using Student’s *t*-test and one-way ANOVA. Significant differences are interpreted for *p* values ≤ 0.05. *p*-values were obtained from pairwise comparisons between treatments or among the three groups.

Next, we analyzed the effects of the modified residues in virus replication, cell-to-cell movement, and systemic spread. To evaluate the effects in virus replication, we quantified the AMV RNA accumulation on P12 protoplasts at 16 h post-transfection. Although Northern blot analysis revealed that the AMV derivative (Figure 4A) carrying the MP^S259F^ accumulated a slightly higher amount of AMV RNA than constructs carrying MP^WT^ or MP^S72F^ (Figure 4B), the *t*-test and ANOVA analyses do not show significant differences (*p* values > 0.05) in viral accumulation among them (Figure 4B).

### 3.3. The Point Mutation S259F Favors a More Efficient Cell-to-Cell Transport

**Figure 5:**
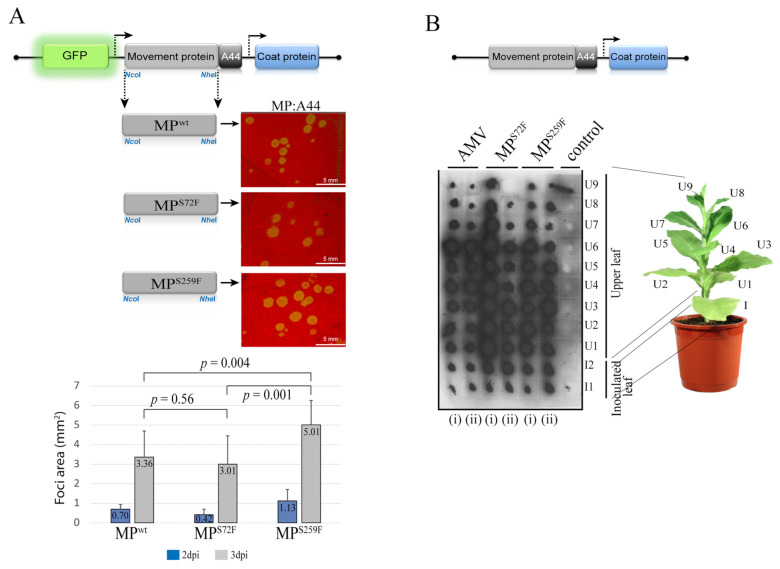
The presence of a phenylalanine at position 259 in the CiLV-C2 MP favors cell-to-cell transport. Analysis of the cell-to-cell and systemic transport of the hybrid AMV RNA 3 constructs carrying the CiLV-C2 MP wild type gene (MP^WT^) or its MP^S72F^ and MP^S259F^ mutants. (**A**) Infection foci observed in P12 plants inoculated with RNA 3 transcripts from pGFP/A255/CP derivatives carrying the heterologous MPs fused to the C-terminal 44 residues of the AMV MP (A44). The schematic representation shows the GFP/A255/CP AMV RNA 3 construct [36], in which the open reading frames, represented by large boxes, correspond to the green fluorescent protein (GFP), the movement protein (MP), and the coat protein (CP). The short box corresponds to the A44, meanwhile, arrows represent subgenomic promoters. The *Nco*I and *Nhe*I restriction sites used to exchange the MPs are indicated. White bars correspond to 5 mm. Histograms represent the average of the area in mm^2^ of 40 independent infection foci at 2 and 3 days post-inoculation (dpi). Error bars indicate the standard deviation. Student’s *t*-test and statistical significance were set at *p* ≤ 0.05. The *p*-values obtained from the comparison between pairs of groups are presented. (**B**) Tissue-printing analysis of P12 plants in duplicate (i and ii) and inoculated with the AMV RNA 3 derivatives showed in (**A**) but lacking the 5′-proximal GFP gene. Plants were analyzed at 14 dpi by printing the transversal section of the corresponding petiole from inoculated (I) and upper (U) leaves. Control corresponds to a healthy plant. This assay was repeated three times.

Next, viral cell-to-cell spread was evaluated by inoculation of P12 leaves with AMV RNA 3 transcripts carrying GFP and the wild type or mutated versions of the CiLV-C2 MP. The area measurement at 3 dpi of 40 individual infection foci showed that the AMV construct carrying the MP^S259F^ generates a more efficient cell-to-cell movement (average of 5.01 mm^2^) than the MP^WT^ (3.36 mm^2^) and the MP^S72F^ (3.01 mm^2^) constructs (*p* values ≤ 0.05), while no significant difference was observed between AMV variants carrying the MP^WT^ and MP^S72F^ (Figure 5A).

Finally, the distribution of the AMV RNA 3 expressing the heterologous MPs in inoculated and systemic P12 leaves was analyzed by tissue-printing of the transversal section of the corresponding petiole at 14 dpi. Positive hybridization signal correlated with the presence of the virus in the corresponding leaf, as previously described [12,46,47]. Despite the clear differences observed for cell-to-cell movement, both CiLV-C2 MP variants (MP^S72F^ and MP^S259F^) exhibited systemic movement similar to that observed for the control (AMV WT), where the presence of viral RNA was detected in all inoculated (I) and upper (U) leaves, in which the petiole was printed on the membrane (Figure 5B). This result was consistent for all replicates.

Taken together, these findings indicate that the point mutation in residue 259 of the CiLV-C2 MP is sufficient to increase local transport but does not affect the systemic movement in the AMV background context and that local movement efficiency is not associated with the viral replication.

### 3.4. CiLV-C2^WT^ vs. Mutated MPs

Given the increased efficiency on cell-to-cell movement observed for the CiLV-C2 MP^S259F^ mutant, we decided to tackle whether this positive effect represents a fitness advantage when compared to the parental CiLV-C2 MP^WT^. Therefore, we performed a competition assay between CiLV-C2 MP^WT^ and either MP^S72F^ or MP^S259F^ chimeric constructs. An equal amount of transcripts derived from the different AMV RNA 3 constructs were mixed and inoculated on P12 plants. Direct sequencing of the MP RT-PCR amplicons from AMV RNA 3 progeny obtained from upper non-inoculated leaves of infected P12 plants (two replicates) revealed that during the first passage, a mix of progeny was observed in all plants/combinations assayed (Table 1). However, after the second serial passage onwards, the AMV population harboring the CiLV-C2 MP^WT^ prevailed over the viral population expressing the MP^S72F^ (Table 1). On the other hand, the viral population containing the MP^S259F^ prevailed in the mixed infection when co-inoculated with the AMV population expressing the CiLV-C2 MP^WT^ (Table 1). These findings were consistent in all replicates. This indicates that the point mutation that generates advantages in cell-to-cell movement could determine the predominance of one viral population over another.

## 4. Discussion

The competition among viral populations may result in the selection of more efficient viral strains, representing a common process in viral evolution [48,49,50]. Here we have shown that an AMV viral construct containing the CiLV-C2 MP prevailed over a similar AMV derivative carrying the CiLV-C MP in competition experiments. It is tempting to hypothesize that the MP may be an important factor associated with the current scenario of cileviruses infecting citrus in Colombia, where CiLV-C2 seems to be replacing CiLV-C in the field [14,16,17,18]. The evolutionary premise that viruses that move more efficiently within their hosts are more likely to be accessible for the transmitting vector could be applicable in many pathosystems, but only partially in those involving cileviruses, since they are unable to colonize their hosts systemically [14]. In this scenario, it is more likely that other factors (e.g., efficiency in viral replication, cell-to-cell transport, virus influences on vector behavior, and vector transmissibility), could be regulating the viral “dispute” in citrus orchards. Nevertheless, further assays are required to address whether or not the MP plays a role in this process under natural conditions.

In addition to the main biological function of viral MPs, which consists in facilitating the infection at the local and systemic viral spread, viral MPs may also play important roles in symptom development and host susceptibility [20,21,22,23,24]. In the present work, studying the CiLV-C2 MP in AMV infectious context, we identified amino acid residues in the MP involved in viral transport. Two spontaneous mutations of a serine residue at positions 72 and 259 to phenylalanine (S^72^ → F and S^259^ → F), in the CiLV-C2 MP gene products, were recovered from P12 tobacco plant from the second passage in the viral fitness assay. We observed that the MP mutation S^259^ → F generated more efficient cell-to-cell movement than MP^WT^ and MP mutation S^72^ → F.

**Figure 6:**
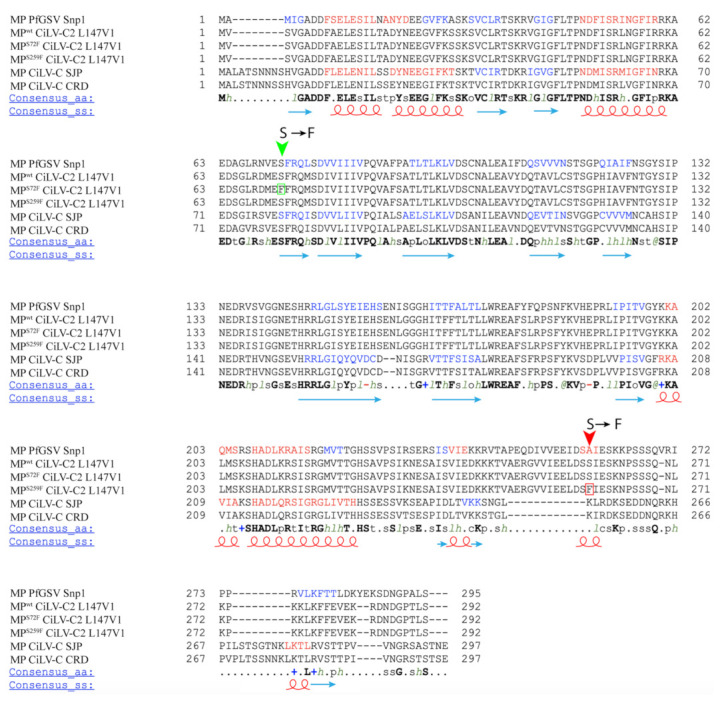
Alignment of the MPs of CiLV-C strain CRD, CiLV-C strain SJP, CiLV-C2 strain L147V1, and passion fruit green spot virus (PfGSV) strain Snp1 using the PROMALS3D program. The annotated secondary structural features were predicted using the PSIPRED algorithm through consensus predictions (red: alpha-helix and blue: beta-strand also represented by red spirals and blue arrows in consensus s.s). The last line shows the consensus amino acids (consensus a.a). Conserved amino acids are in bold and capital letters. Symbols: I, aliphatic; @, aromatic; h, hydrophobic; o, alcohol; p, polar residues; t, tiny; s, small; b, bulky residues; +, positively charged; -, negatively charged; c, charged. The amino acids mutated are highlighted by the green (S → F, MP^S72F^) and red (S → F, MP^S259F^) arrows. The GenBank accession numbers of the cilevirus MPs used for this analysis are CiLV-C strain CRD (YP_654542.1), CiLV-C strain SJP (AKJ79137.1), CiLV-C2 strain L147V1 (AGE82893.1), and PfGSV strain Snp1 (MK804172.1).

**Figure 7:**
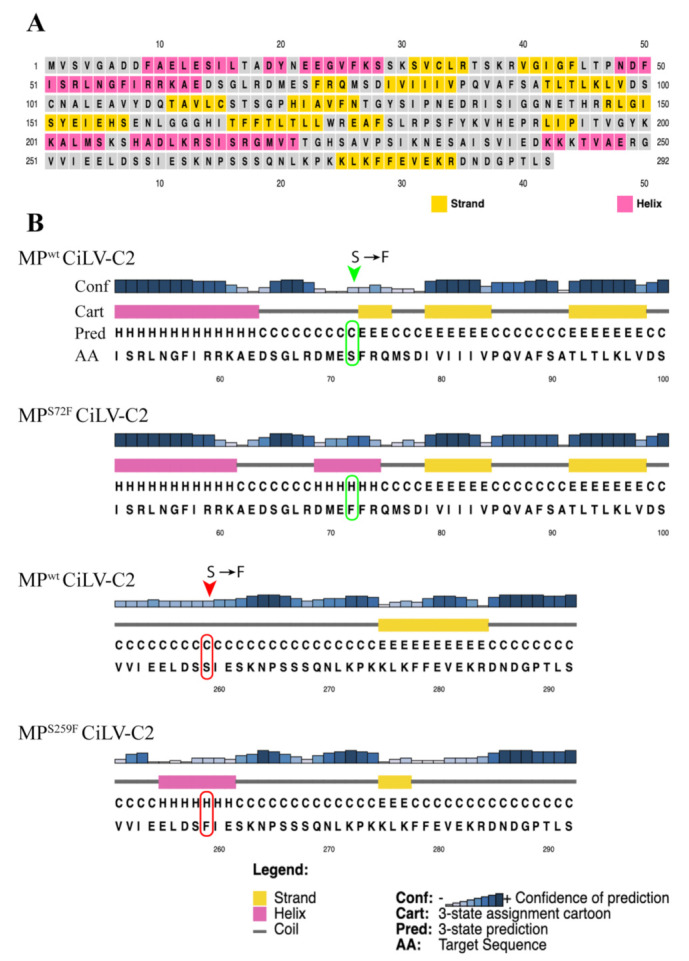
Annotated secondary structures of the CiLV-C2 MP^WT^, MP^S72F^, and MP^S259F^. (**A**) Sequence plot showing a global map of the secondary structures present in the CiLV-C2 MP^WT^. (**B**) PSIPRED cartoons show a comparison of secondary structures between CiLV-C2 MP^WT^ with MP^S72F^ and MP^S259F^ in the MP regions (N- and C-terminus) where the mutations are present. The amino acids mutated are highlighted by the green (S → F, MP^S72F^) and red (S → F, MP^S259F^) arrows. The GenBank accession number of the CiLV-C2 strain L147V1 is AGE82893.1.

It has been previously observed that amino acid changes in MP residues mainly affect the functional aspects associated with the viral cell-to-cell and systemic transport [22,26,27]. The mutation S^259^ → F that spontaneously appeared after the second passage in competition experiments affected positively the AMV cell-to-cell movement and had no effects on systemic movement. To better understand the changes in the MP sequence with improved cell-to-cell movement, we performed an amino acid alignment among the sequences of representative cileviruses available in the GenBank (CiLV-C strain CRD, CiLV-C strain SJP, CiLV-C2 strain L147V1, and PfGSV strain Snp1), using the profile multiple alignment sequence with predicated local structures and 3D constraints (PROMALS3D) program [43,44] (Figure 6). Among the two mutations showed here, only the N-terminal-proximal mutation S^72^ → F was identified into the central core region of the 30K superfamily members [6,8], between amino acids 56 and 229 in the cilevirus MPs, more specifically into the first β-structure. It is important to note that the amino acid serine at position 72 is conserved among all cilevirus MP sequences. To gain additional insights about the influence of the mutations in protein folding, we evaluated the secondary structures of mutated MPs compared to MP^WT^ using the PSIPRED tool (http://bioinf.cs.ucl.ac.uk/psipred/, accessed on 5 December 2021) (Figure 7). Even though a polar amino acid was changed by a nonpolar residue, resulting in a change from beta-strand to alpha-helix structure (Figure 7B), we speculate that this change to phenylalanine had an effect very moderate, since both MP^WT^ and MP^S72F^ proteins behave similarly in the different analyzed viral functions. However, the fitness analysis revealed the prevalence of the MP^WT^ suggesting that CiLV-C2 MP has evolved to avoid the changes in this conserved residue through a smooth fitness penalty, as reported for other pathosystems [28,51].

Unlike the CiLV-C2 MP^S72F^, the mutation of the S residue at position 259 to phenylalanine generated an MP more efficient than the MP^WT^, at least for local transport. The presence of a phenylalanine also resulted in a putative change in the protein folding but, in this case, the appearance of a helix structure in the mutated region is predicted (Figure 7B). This amino acid is not conserved between all cilevirus MPs sequences analyzed herein, probably due to being located in the more variable C-terminal region (Figure 6). The C-terminus of the MPs assigned to the 30K superfamily has been described to be a multifunctional region intrinsically implicated with several aspects of viral movement, such as interaction with the cognate capsid protein (CP), formation of tubular structures, and efficiency in viral cell-to-cell and systemic transport [10,13,46,52,53]. In our recent study, the sequential C-terminal deletion of CiLV-C2 MP showed that, although the absence of this fragment (deletion of 70 residues) still enables cell-to-cell movement, its removal impairs the correct tubule polymerization and MP-plasmodesma association, affecting the viral cell-to-cell and long-distance transport; furthermore, this region is independently responsible to recruit the p29 (cilevirus capsid protein) to the cell periphery [34]. Consequently, any MP modification in this region could affect one or several viral movement processes. Given the positive effect in AMV transport by this amino acid change, it would be interesting to assess whether the presence of a putative alpha-helix structure at the carboxy-terminal of CiLV-C2 MP could favor the tubule formation and/or the MP association with plasmodesmata, since it has been shown that these processes were essential for an efficient AMV transport mediated by cilevirus MP [34].

In theory, during the competition between viral populations, the virus strain which first reaches the vascular tissue would be more likely to spread throughout the plant. CiLV-C MP generates a more efficient cell-to-cell movement but also a more inefficient systemic transport and less accumulation of vRNA than CiLV-C2 MP. The competition analysis between both MPs revealed the prevalence of the AMV derivative carrying the CiLV-C2 MP, indicating that the systemic movement over the local transport is the determinant factor for the selection of a viral isolate in the plant. However, when no differences in the efficiency in systemic movement between two viral variants (MP^S259F^ vs. MP^WT^) exist, we observed that the variant that generates the most efficient local movement was the prevalent one, indicating that local movement also has an effect on the prevalence of a viral isolate but is subjected to the systematic transport. Moreover, there was no difference in viral replication between populations carrying CiLV-C2 MP variants, being that the one with the best movement efficiency was the most prevalent. Thus, we can speculate that in the context of AMV infection, viral accumulation is not an essential factor for the selection of a viral isolate in the plant.

In summary, in this study, we present evidence that mutations in the MP gene that increased the viral cell-to-cell movement directly correlated with a higher viral fitness. Thereby, our findings can help to explain in which biological processes of the viral infection cycle a genetic mutation could generate evolutionary benefits.

## Figures and Tables

**Table 1:** Viral fitness assay between the AMV RNA 3 constructs carrying the CiLV-C2 MP wild type (MP^WT^) and its MP^S72F^ or MP^S259F^ mutants.

Viral Inoculum Passage	Repetition	MP^WT^ vs. MP^S72F^	MP^WT^ vs. MP^S259F^
1st passage	plant 1	MP^WT^/MP^S72F^	MP^WT^/MP^S259F^
	plant 2	MP^WT^/MP^S72F^	MP^WT^/MP^S259F^
2nd passage	plant 1	MP^WT^	MP^S259F^
	plant 2	MP^WT^	MP^S259F^
3rd passage	plant 1	MP^WT^	MP^S259F^
	plant 2	MP^WT^	MP^S259F^

## Data Availability

All virus sequences are available on NCBI (accession numbers: YP_654542.1, AKJ79137.1, AGE82893.1, MK804172.1).

## Supplementary Materials

The following are available online at https://www.mdpi.com/article/10.3390/v13122498/s1, Figure S1: Analysis of the cell-to-cell and systemic transport of the hybrid AMV RNA 3 constructs carrying the CiLV-C and CiLV-C2 MPs, Figures S2–S6: Full-length gels and blots from Figure 1B, Figure 2, Figure 4B, Figure 5B and Figure S1.

## References

[1] Dorokhov Y.L., Sheshukova E.V., Byalik T.E., Komarova T.V. (2020). Diversity of Plant Virus Movement Proteins: What Do They Have in Common?. Processes.

[2] Heinlein M., Epel B.L. (2004). Macromolecular Transport and Signaling Through Plasmodesmata. Int. Rev. Cytol..

[3] Ding B. (1998). Intercellular protein trafficking through plasmodesmata. Protein Trafficking in Plant Cells.

[4] Lucas W.J. (2006). Plant viral movement proteins: Agents for cell-to-cell trafficking of viral genomes. Virology.

[5] Navarro J.A., Sanchez-Navarro J.A., Pallas V. (2019). Key checkpoints in the movement of plant viruses through the host. Adv. Virus Res..

[6] Melcher U. (2000). The ‘30K’ superfamily of viral movement proteins. J. Gen. Virol..

[7] Niehl A., Heinlein M. (2011). Cellular pathways for viral transport through plasmodesmata. Protoplasma.

[8] Mushegian A.R., Elena S.F. (2015). Evolution of plant virus movement proteins from the 30K superfamily and of their homologs integrated in plant genomes. Virology.

[9] Taliansky M., Torrance L., Kalinina N.O. (2008). Role of Plant Virus Movement Proteins. Methods Mol. Biol..

[10] Lewandowski D.J., Adkins S. (2005). The tubule-forming NSm protein from Tomato spotted wilt virus complements cell-to-cell and long-distance movement of Tobacco mosaic virus hybrids. Virology.

[11] Wolf S., Lucas W.J., Deom C.M., Beachy R.N. (1989). Movement Protein of Tobacco Mosaic Virus Modifies Plasmodesmatal Size Exclusion Limit. Science.

[12] Fajardo T.V.M., Peiró A., Pallás V., Sanchez-Navarro J.A. (2013). Systemic transport of Alfalfa mosaic virus can be mediated by the movement proteins of several viruses assigned to five genera of the 30K family. J. Gen. Virol..

[13] Sanchez-Navarro J.A., Carmen Herranz M., Pallas V. (2006). Cell-to-cell movement of Alfalfa mosaic virus can be mediated by the movement proteins of Ilar-, bromo-, cucumo-, tobamo- and comoviruses and does not require virion formation. Virology.

[14] Freitas-Astua J., Ramos-Gonzalez P.L., Arena G.D., Tassi A.D., Kitajima E.W. (2018). Brevipalpus-transmitted viruses: Parallelism beyond a common vector or convergent evolution of distantly related pathogens?. Curr. Opin. Virol..

[15] Bastianel M., Novelli V.M., Kitajima E.W., Kubo K.S., Bassanezi R.B., Machado M.A., Freitas-Astúa J. (2010). Citrus Leprosis: Centennial of an Unusual Mite–Virus Pathosystem. Plant Dis..

[16] Roy A., Choudhary N., Guillermo L.M., Shao J., Govindarajulu A., Achor D., Wei G., Picton D.D., Levy L., Nakhla M.K. (2013). A Novel Virus of the Genus Cilevirus Causing Symptoms Similar to Citrus Leprosis. Phytopathology.

[17] Roy A., Hartung J.S., Schneider W.L., Shao J., Leon G., Melzer M.J., Beard J.J., Otero-Colina G., Bauchan G.R., Ochoa R. (2015). Role Bending: Complex Relationships Between Viruses, Hosts, and Vectors Related to Citrus Leprosis, an Emerging Disease. Phytopathology.

[18] Leon M.G., Becerra C.H., Freitas-Astua J., Salaroli R.B., Kitajima E.W. (2008). Natural Infection of Swinglea glutinosa by the Citrus leprosis virus Cytoplasmic Type (CiLV-C) in Colombia. Plant Dis..

[19] Leastro M.O., Freitas-Astúa J., Kitajima E.W., Pallás V., Sánchez-Navarro J.A. (2021). Membrane Association and Topology of Citrus Leprosis Virus C2 Movement and Capsid Proteins. Microorganisms.

[20] Pallas V., Garcia J.A. (2011). How do plant viruses induce disease? Interactions and interference with host components. J. Gen. Virol..

[21] Li W., Lewandowski D.J., Hilf M.E., Adkins S. (2009). Identification of domains of the Tomato spotted wilt virus NSm protein involved in tubule formation, movement and symptomatology. Virology.

[22] Margaria P., Anderson C.T., Turina M., Rosa C. (2016). Identification of Ourmiavirus 30K movement protein amino acid residues involved in symptomatology, viral movement, subcellular localization and tubule formation. Mol. Plant Pathol..

[23] García J.A., Pallás V. (2015). Viral factors involved in plant pathogenesis. Curr. Opin. Virol..

[24] Takeshita M., Suzuki M., Takanami Y. (2001). Combination of amino acids in the 3a protein and the coat protein of Cucumber mosaic virus determines symptom expression and viral spread in bottle gourd. Arch. Virol..

[25] Zhao W., Ji Y., Wu S., Ma X., Li S., Sun F., Cheng Z., Zhou Y., Fan Y. (2018). Single amino acid in V2 encoded by TYLCV is responsible for its self-interaction, aggregates and pathogenicity. Sci. Rep..

[26] Wobbe K.K., Akgoz M., Dempsey D.A., Klessig D.F. (1998). A Single Amino Acid Change in Turnip Crinkle Virus Movement Protein p8 Affects RNA Binding and Virulence on Arabidopsis thaliana. J. Virol..

[27] Choi S.K., Palukaitis P., Min B.E., Lee M.Y., Choi J.K., Ryu K.H. (2005). Cucumber mosaic virus 2a polymerase and 3a movement proteins independently affect both virus movement and the timing of symptom development in zucchini squash. J. Gen. Virol..

[28] Peiro A., Canizares M.C., Rubio L., Lopez C., Moriones E., Aramburu J., Sanchez-Navarro J. (2014). The movement protein (NSm) of Tomato spotted wilt virus is the avirulence determinant in the tomato Sw-5 gene-based resistance. Mol. Plant Pathol..

[29] Wang H.-L., Wang Y., Giesman-Cookmeyer D., Lommel S.A., Lucas W.J. (1998). Mutations in Viral Movement Protein Alter Systemic Infection and Identify an Intercellular Barrier to Entry into the Phloem Long-Distance Transport System. Virology.

[30] Wieczorek P., Obrępalska-Stęplowska A. (2016). A single amino acid substitution in movement protein of tomato torrado virus influences ToTV infectivity in Solanum lycopersicum. Virus Res..

[31] Leastro M.O., Kitajima E.W., Silva M.S., Resende R.O., Freitas-Astúa J. (2018). Dissecting the Subcellular Localization, Intracellular Trafficking, Interactions, Membrane Association, and Topology of Citrus Leprosis Virus C Proteins. Front. Plant Sci..

[32] Locali-Fabris E.C., Freitas-Astua J., Souza A.A., Takita M.A., Astúa-Monge G., Antonioli-Luizon R., Rodrigues V., Targon M.L.P.N., Machado M.A. (2006). Complete nucleotide sequence, genomic organization and phylogenetic analysis of Citrus leprosis virus cytoplasmic type. J. Gen. Virol..

[33] Pascon R.C., Kitajima J.P., Breton M.C., Assumpcao L., Greggio C., Zanca A.S., Okura V.K., Alegria M.C., Camargo M.E., Silva G.G. (2006). The complete nucleotide sequence and genomic organization of Citrus Leprosis associated Virus, Cytoplasmatic type (CiLV-C). Virus Genes.

[34] Leastro M.O., Freitas-Astúa J., Kitajima E.W., Pallás V., Sánchez-Navarro J.A. (2021). Unravelling the involvement of cilevirus p32 protein in the viral transport. Sci. Rep..

[35] Van Dun C.M., van Vloten-Doting L., Bol J.F. (1988). Expression of alfalfa mosaic virus cDNA1 and 2 in transgenic tobacco plants. Virology.

[36] Sanchez-Navarro J.A., Miglino R., Ragozzino A., Bol J.F. (2001). Engineering of Alfalfa mosaic virus RNA 3 into an expression vector. Arch. Virol..

[37] Aparicio F., Pallas V., Sanchez-Navarro J.A. (2010). Implication of the C terminus of the Prunus necrotic ringspot virus movement protein in cell-to-cell transport and in its interaction with the coat protein. J. Gen. Virol..

[38] Taschner P.E., Van Der Kuyl A.C., Neeleman L., Bol J.F. (1991). Replication of an incomplete alfalfa mosaic virus genome in plants transformed with viral replicase genes. Virology.

[39] Loesch-Fries L.S., Jarvis N.P., Krahn K.J., Nelson S.E., Hall T.C. (1985). Expression of Alfalfa Mosaic virus RNA 4 cDNA transcripts in Vitro and in Vivo. Virology.

[40] Pallás V., Más P., Sánchez-Navarro J.A. (1998). Detection of Plant RNA Viruses by Nonisotopic Dot-Blot Hybridization. Methods Mol. Biol..

[41] Saánchez-Navarro J., Fajardo T., Zicca S., Pallaás V., Stavolone L. (2010). Caulimoviridae Tubule-Guided Transport Is Dictated by Movement Protein Properties. J. Virol..

[42] Leastro M.O., Castro D.Y.O., Freitas-Astua J., Kitajima E.W., Pallás V., Sánchez-Navarro J. (2020). Citrus Leprosis Virus C Encodes Three Proteins with Gene Silencing Suppression Activity. Front. Microbiol..

[43] Pei J., Grishin N.V. (2014). PROMALS3D: Multiple protein sequence alignment enhanced with evolutionary and three-dimensional structural information. Methods Mol. Biol..

[44] Jones D.T. (1999). Protein secondary structure prediction based on position-specific scoring matrices. J. Mol. Biol..

[45] Leastro M.O., Freitas-Astúa J., Kitajima E.W., Pallás V., Sánchez-Navarro J.A. (2020). Dichorhaviruses Movement Protein and Nucleoprotein Form a Protein Complex That May Be Required for Virus Spread and Interacts in vivo With Viral Movement-Related Cilevirus Proteins. Front. Microbiol..

[46] Leastro M.O., Pallas V., Resende R.O., Sanchez-Navarro J.A. (2017). The functional analysis of distinct tospovirus movement proteins (NSM) reveals different capabilities in tubule formation, cell-to-cell and systemic virus movement among the tospovirus species. Virus Res..

[47] Mas P., Pallás V. (1995). Non-isotopic tissue-printing hybridization: A new technique to study long-distance plant virus movement. J. Virol. Methods.

[48] Power A.G. (1996). Competition between Viruses in a Complex Plant--Pathogen System. Ecology.

[49] Amaku M., Burattini M.N., Coutinho F.A.B., Massad E. (2010). Modeling the Competition Between Viruses in a Complex Plant–Pathogen System. Phytopathology.

[50] Amaku M., Burattini M.N., Coutinho F.A.B., Massad E. (2010). Modeling the Dynamics of Viral Evolution Considering Competition Within Individual Hosts and at Population Level: The Effects of Treatment. Bull. Math. Biol..

[51] Khatabi B., Wen R.-H., Hajimorad M.R. (2013). Fitness penalty in susceptible host is associated with virulence of Soybean mosaic virus on Rsv1-genotype soybean: A consequence of perturbation of HC-Pro and not P3. Mol. Plant Pathol..

[52] Sanchez-Navarro J.A., Bol J.F. (2001). Role of the Alfalfa mosaic virus Movement Protein and Coat Protein in Virus Transport. Mol. Plant-Microbe Interact..

[53] Nagano H., Okuno T., Mise K., Furusawa I. (1997). Deletion of the C-terminal 33 amino acids of cucumber mosaic virus movement protein enables a chimeric brome mosaic virus to move from cell to cell. J. Virol..

