# Supplementary material for: Spontaneous Mutation in the Movement Protein of Citrus Leprosis Virus C2, in a Heterologous Virus Infection Context, Increases Cell-to-Cell Transport and Generates Fitness Advantage
Source: Viruses. 2021 Dec 13;13(12):2498. doi: 10.3390/v13122498 (PMC8708801; doi:10.3390/v13122498)
Supplement: Supplementary file 1 [file viruses-13-02498-s001.zip › viruses-1455924-supplementary.pdf]

viruses

MDPI

Supplementary Materials

Spontaneous Mutation in the Movement Protein of Citrus Leprosis Virus C2, in a Heterologous Virus Infection Context, Increases Cell-to-Cell Transport and Generates Fitness Advantage

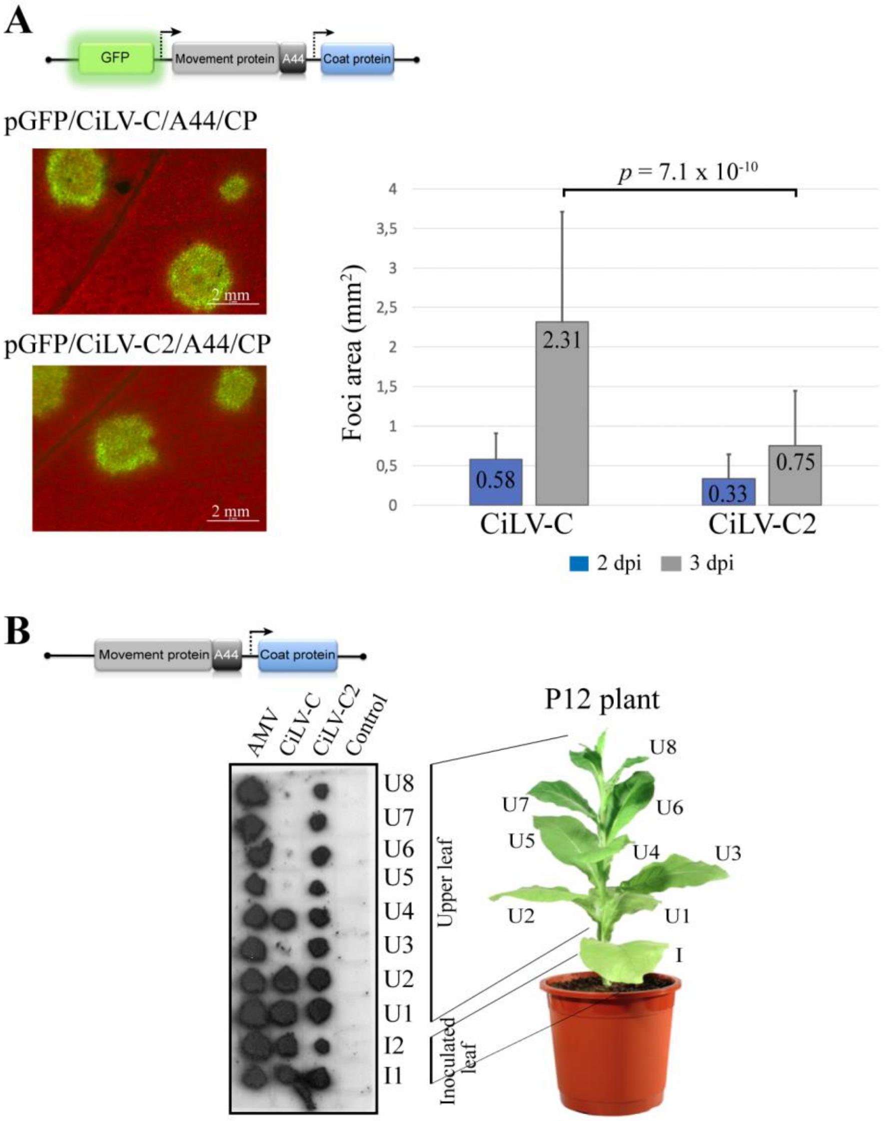

Figure S1. Analysis of the cell-to-cell and systemic transport of the hybrid AMV RNA 3 constructs carrying the CiLV-C and CiLV-C2 MPs. This results were adapted from Leastro, et al. [1] (**A**) Infection foci observed in P12 plants inoculated with RNA 3 transcripts from pGFP/A255/CP derivatives carrying the heterologous MPs containing stop codon, thus not fused to the C-terminal 44 residues of the AMV MP (A44). The schematic representation shows the GFP/A255/CP AMV RNA 3 construct [2], in which the open reading frames, represented by large boxes, correspond to the green fluorescent protein (GFP), the movement protein (MP), and the coat protein (CP). The short box corresponds to the A44, meanwhile arrows represent subgenomic promoters. White bars correspond to 2 mm. Histograms represent the average of the area in mm^2 of 40 independent infection foci at 2 and 3 days post-inoculation (dpi). Error bars indicate the standard deviation. Student's *t*-test and statistical significance was set at $p \leq 0.05$. The *p*-values obtained from comparison between pairs of groups are presented. (**B**) Tissue-printing analysis of P12 plants inoculated with the AMV RNA 3 derivatives showed in (A), but lacking the 5′ proximal GFP gene. Plants were analyzed at 14

dpi by printing the transversal section of the corresponding petiole from inoculated (I) and upper (U) leaves. Control, corresponds to healthy plant. This assay was repeated three times.

Supporting Information files: Full-length gels and blots

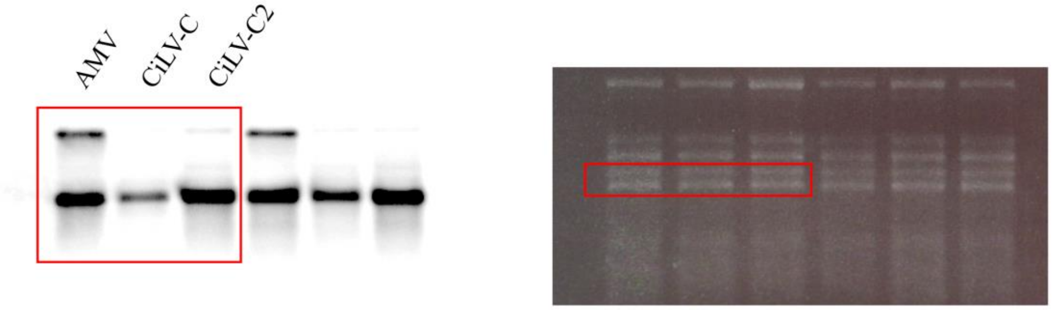

Figure S2. Northern blot analysis from figure 1B. The regions of the original blot and gel used in main figures were denoted by red boxes.

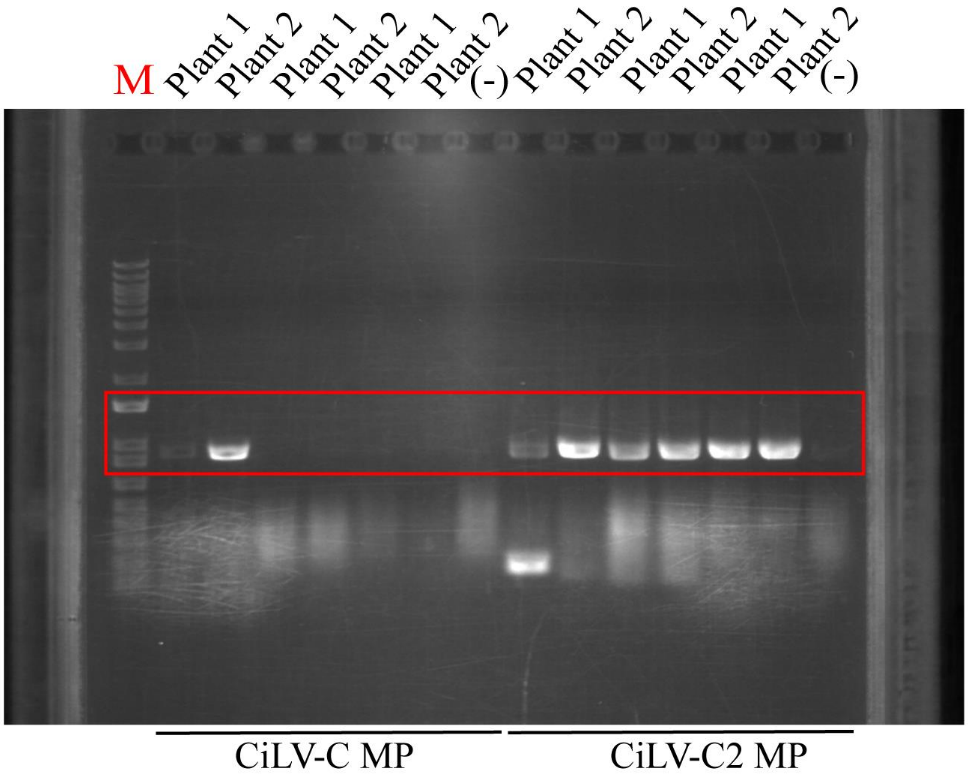

Figure S3. Gel from competition analysis presented in the figure 2. The region of the original gel used in main figure was denoted by red box.

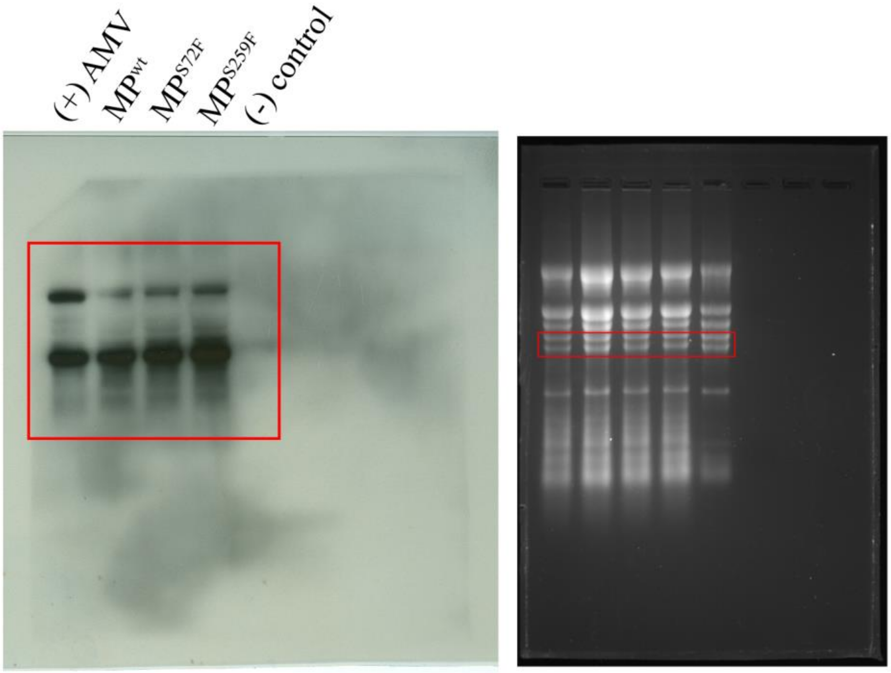

Figure S4. Northern blot analysis from figure 4B. The regions of the original blot and gel used in main figure were denoted by red boxes.

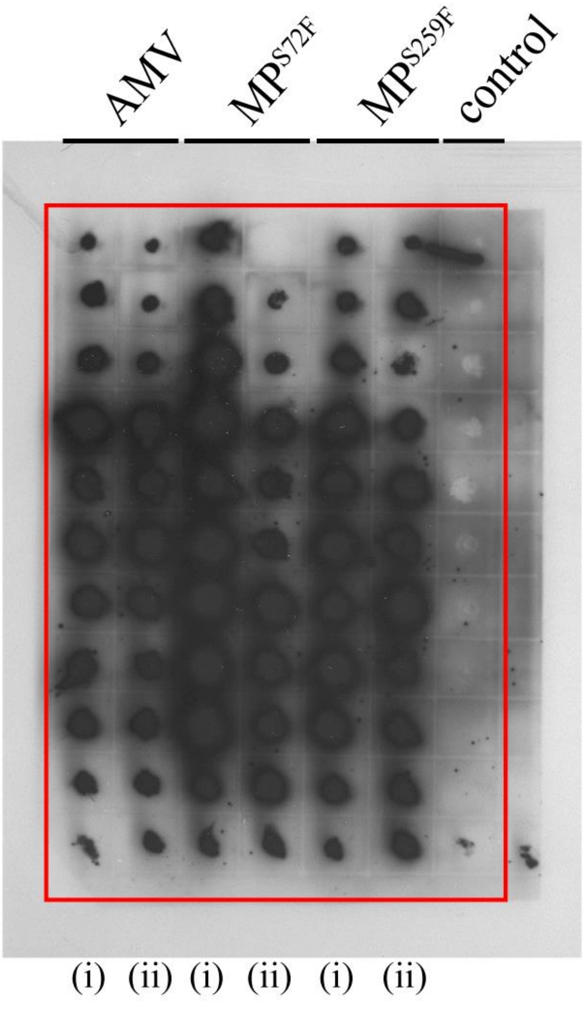

Figure S5. Tissue print from figure 5B. The region of the original blot used in main figure was denoted by red box.

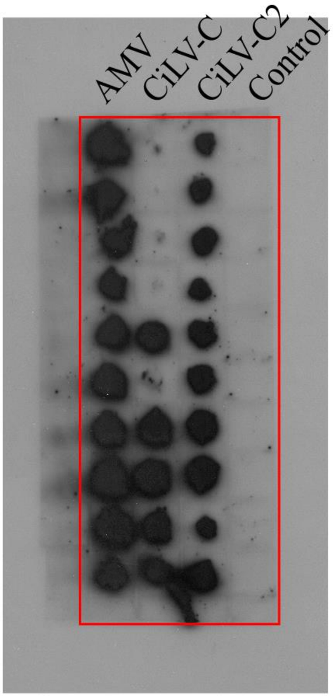

Figure S6. Tissue print from figure S1. The region of the original blot used in main figure was denoted by red box. .
